# A deep transfer learning approach for wearable sleep stage classification with photoplethysmography

**DOI:** 10.1038/s41746-021-00510-8

**Published:** 2021-09-15

**Authors:** Mustafa Radha, Pedro Fonseca, Arnaud Moreau, Marco Ross, Andreas Cerny, Peter Anderer, Xi Long, Ronald M. Aarts

**Affiliations:** 1 Philips Research, Eindhoven, the Netherlands; 2 Department of Electrical Engineering, Eindhoven University of Technology, Eindhoven, the Netherlands; 3 Philips Austria GmbH, Vienna, Austria

**Keywords:** Health care, Signs and symptoms

## Abstract

Unobtrusive home sleep monitoring using wrist-worn wearable photoplethysmography (PPG) could open the way for better sleep disorder screening and health monitoring. However, PPG is rarely included in large sleep studies with gold-standard sleep annotation from polysomnography. Therefore, training data-intensive state-of-the-art deep neural networks is challenging. In this work a deep recurrent neural network is first trained using a large sleep data set with electrocardiogram (ECG) data (292 participants, 584 recordings) to perform 4-class sleep stage classification (wake, rapid-eye-movement, N1/N2, and N3). A small part of its weights is adapted to a smaller, newer PPG data set (60 healthy participants, 101 recordings) through three variations of transfer learning. Best results (Cohen’s kappa of 0.65 ± 0.11, accuracy of 76.36 ± 7.57%) were achieved with the domain and decision combined transfer learning strategy, significantly outperforming the PPG-trained and ECG-trained baselines. This performance for PPG-based 4-class sleep stage classification is unprecedented in literature, bringing home sleep stage monitoring closer to clinical use. The work demonstrates the merit of transfer learning in developing reliable methods for new sensor technologies by reusing similar, older non-wearable data sets. Further study should evaluate our approach in patients with sleep disorders such as insomnia and sleep apnoea.

## Introduction

The objective measurement of sleep at home in an unobtrusive manner has become an increasingly important topic of study as systematic sleep deprivation is increasingly linked to health adversities such as weight gain^1^, systemic inflammation^2^, weakened glucose regulation^3^ and poor fitness to drive^4^. Next to that, sleep assessments in sleep medicine could also benefit from longitudinal home monitoring^5^, which could provide a complementary role to gold-standard polysomnography (PSG) measurements. The introduction of actigraphy into clinical sleep assessment has allowed a different perspective on patient’s conditions^6^, even though it can only be used to automatically approximate sleep-wake patterns and cannot reliably distinguish between the different stages of sleep: rapid-eye-movement (REM) sleep and the three levels of non-REM sleep (N1, N2, N3)^7^. A method which could automatically detect these sleep stages at home could further advance home sleep assessment.

**Fig. 1:**
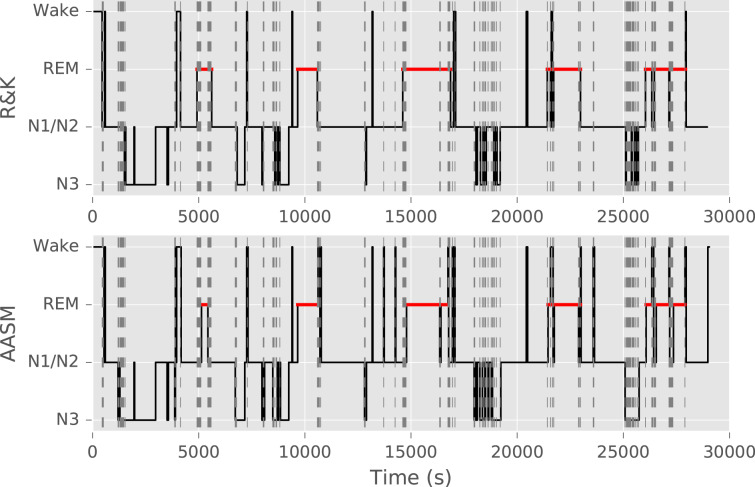
Sleep hypnogram from the Siesta database simultaneously annotated according to R&K and AASM annotation standards. Due to differences in annotation rules, a total of 59 min of this night were differently annotated (differences highlighted with vertical grey stripes). Note that some changes may also be related to inter-rater disagreement. For ease of visual comparison, instead of presenting full hypnograms, this figure shows only 4-class hypnograms (Wake, REM sleep, N1/N2 sleep, and N3 sleep), which is also the objective of automatic sleep stage classification in this study.

Sleep stage scoring is normally done through manual visual annotation of PSG data, which include electro-graphic measurements of cortical brain activity as well as eye and chin muscle activity. Every 30 s epoch (i.e. segment) of sleep is labelled as one of the sleep stages or as wake. The resulting annotation for the entire night is referred to as a sleep hypnogram^8^. Sample hypnograms are illustrated in Fig. 1. The current scoring guidelines are maintained by the American Academy of Sleep Medicine (AASM)^8^. However, before 2007 the Rechtschaffen & Kales (R&K)^9^ guidelines were the most commonly used, since their publication in 1968. While these standards are very comparable, structural differences have been found that lead to different results when comparing AASM to R&K annotation: increase in N1 and N3 scoring; a decrease in N2 scoring; and a decrease in REM scoring in younger people^10^. An example of a night concurrently scored using both guidelines is illustrated in Fig. 1.

Heart rate variability (HRV) is a heavily studied surrogate to PSG. The autonomic nervous system’s activity is correlated with the progression of sleep stages throughout the night and thus HRV measurements of autonomic activity can be used to estimate sleep stages. The inference of sleep stages from HRV features is done by training machine learning algorithms which translate HRV features to sleep stages. It is still very difficult to perform 5-class sleep stage classification (Wake-REM-N1-N2-N3) due to the low agreement on N1 sleep^11,12^, and thus most recent approaches focus on 4-class sleep stage classification where N1 and N2 are combined (i.e. Wake-REM-N1/2-N3). HRV features are most commonly extracted from ECG as this sensor is part of standard PSG montages and thus it is easy to find data to develop models with^13–15^. The performance of such methods is often measured using the Cohen’s kappa coefficient of inter-rater agreement. This coefficient measures the percentage of epochs where the classifier agrees with the PSG annotation while factoring out agreement by chance that can occur due to class imbalance. Other common metrics such as accuracy or F1 scores are often reported as well.

While methods using ECG data provide a first proof of the feasibility of HRV-based sleep stage classification, the ECG sensor still requires multiple electrodes to be attached to the body, which might limit prolonged use at home. A popular, low-cost and comfortable alternative for measuring HRV is photoplethysmography (PPG), which can be incorporated in wrist band wearables such as smart watches.

Although there were some studies in the past years that used wrist-worn PPG for sleep classification, many of them were proposed with only sleep and wake classes or three classes including wake, REM sleep, and non-REM sleep^16–18^. For sleep-wake classification, important sleep statistics can be evaluated such as total sleep time (TST), sleep efficiency (SE), sleep onset latency (SOL), and wake-after-sleep-onset (WASO). A few methods were evaluated in earlier work for PPG-based 4-class sleep stage classification using traditional machine learning models^19–22^. Recently, deep learning methods, specifically long- and short-term memory (LSTM) models, have shown unprecedented agreement levels with PSG^23–25^. This is thought to be because of the strong temporal learning capabilities of LSTM models^26^, allowing the model to infer the sleep stage over a wide temporal context. For the electro-encephalography (EEG) domain performance levels have been reported that are on par with inter-human annotation agreement^23,24^. Recently, LSTM models also showed a strong improvement over the state-of-the-art for, e.g., radio frequency devices^27^ and ECG^28^. These LSTM models have hundreds of thousands of free model parameters and training them requires larger data sets than traditional machine learning models. Until now, LSTM’s have been barely studied for PPG with a limited improvement in sleep staging^29^, likely due to the lack of large PPG data sets to train such deep models.

**Fig. 2:**
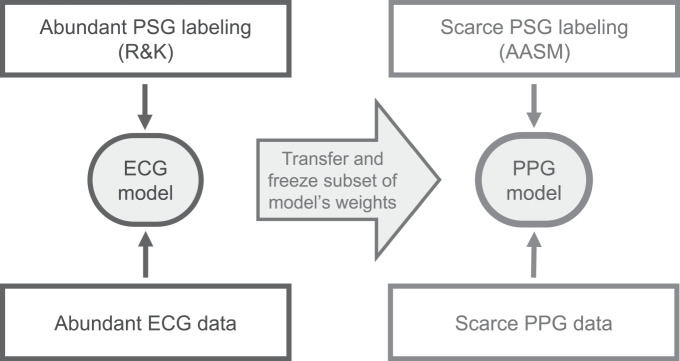
Transfer learning. The source model (ECG model) is trained using ECG data and PSG based labels scored according to the R&K rules (Siesta data set in this work) and then its knowledge is transferred to learn a new task, involving PPG input data and PSG annotation according to the AASM rules (Eindhoven data set in this work), resulting in the PPG model.

Collecting the large data sets required for training deep learning models that contain both PPG and PSG is prohibitively expensive since wrist-worn PPG is not part of standard PSG montages. However, there are strong similarities between HRV derived from PPG and ECG, and there are strong similarities between AASM and R&K annotation. In this work it is proposed to exploit these similarities through transfer learning^30^. The technique intuitively involves transferring knowledge from a model trained on a large “source” data set to solve a new but related problem where less data samples are available in a “target” data set. The process is illustrated in Fig. 2. The change from ECG to PPG is regarded as a domain shift while the change from R&K to AASM scoring is a shift in the target of the machine learning task. This simultaneous shift in domain and target has been called *inductive transfer learning*^30^. Transfer learning has been proven effective in the context of deep neural networks, where knowledge is represented in a modular structure of layers and has been applied successfully in both computer vision^31^ and natural language processing^32^ networks. Notably, Shashikuma et al.^33^ used this technique for cardiac arrhythmia detection, where a model was trained on ECG-derived heart beats and its knowledge was transferred to perform PPG-based cardiac arrhythmia detection, providing evidence for its efficacy in the physiological monitoring domain.

**Fig. 3:**
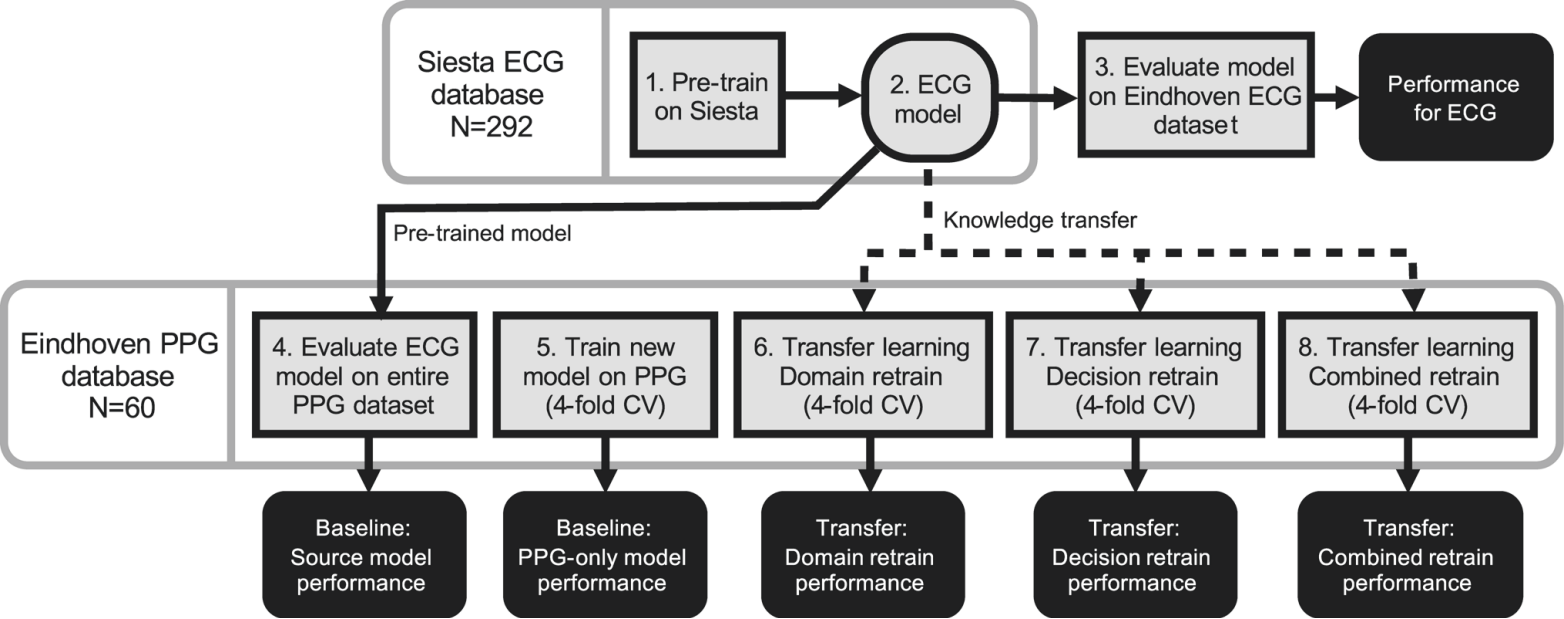
Overview of the validation scheme. The top horizontal lane describes operations done using the Siesta data while the bottom lane describes the validations on the Eindhoven data. Square boxes describe model training operations, rounded black boxes describe endpoints that are statistically compared to confirm the hypothesis of this work, and the rounded grey box denotes the trained ECG model, which is used either as a pre-trained model or adapted via knowledge transfer for PPG-based sleep stage classification as indicated by the arrows flowing out of it. CV means cross-validation across participants.

In this work a transfer learning methodology will be evaluated for transferring the deep temporal knowledge learned using an LSTM model on the ECG data to a newer, smaller data set comprising PPG that is annotated according to new sleep scoring rules. The LSTM network architecture including three main blocks or layers: domain, temporal, and decision (see Methods section). Figure 3 illustrates three transfer learning strategies (*domain*, *decision*, and *combined*) considered in this work. We show that a transfer learning approach, in which a model is first trained on a large ECG data set and then adapted using a small PPG data set, leads to better performance for PPG-based sleep stage classification than when using only ECG data or only PPG data to train the model.

## Results

### Description of demographics and sleep statistics

Two data sets were used to create and validate the approaches. The first data set is Siesta^34^, including 292 participants (584 overnight recordings), a large data set with ECG signals and sleep scoring according to older R&K guidelines from the PSG signals. The second one is the Eindhoven data set^19^, including 60 participants (101 overnight recordings), used for transfer learning to PPG and AASM scoring as it contains both PPG and annotation according to AASM rules from PSG. Table 1 indicates the participant demographics, and corresponding sleep statistics of the two data sets. More information on the two data sets and the corresponding studies are introduced in the Methods section.

**Table 1:** Demographics and sleep statistics of participants in the two data sets used in the study.

Parameter	Siesta data	Eindhoven data
*N*	292 participants (126 females, 43.2%), 584 recordings (252 females, 43.2%)	60 participants (26 females, 43.3%), 101 recordings (48 females, 47.5%)
Age (year)	51.5 (17.3), 20.0−95.0	51.1 (7.9), 41.0−66.0
BMI (kg/m^2^)	25.6 (4.5), 16.5−43.3	25.6 (3.9), 17.5−36.2
TIB (hour)	8.0 (0.5), 5.8−9.6	7.9 (0.7), 6.4−10.3
SE (%)	80.8 (12.8), 14.6−99.1	85.0 (9.8), 36.0−96.6
N1 sleep (%)	13.1 (8.4), 2.4−77.1	10.7 (5.0), 3.0−30.6
N2 sleep (%)	53.8 (8.8), 13.6−78.8	41.7 (8.7), 22.2−66.6
N3 sleep (%)	13.8 (8.4), 0.0−44.5	26.2 (8.7), 10.3−47.3
REM sleep (%)	18.2 (5.9), 0.0−34.8	21.4 (5.9), 9.2−38.2

Note: Sleep statistics are computed based on the sleep stage annotation of the data set. Except for N, results are presented as mean (standard deviation), range. The percentages of N1, N2, N3, and REM sleep were normalized to total sleep time (excluding wake time). *BMI* body mass index, *TIB* time in bed, *SE* sleep efficiency.

### Discrepancy between ECG- and PPG-derived HRV features

**Fig. 4:**
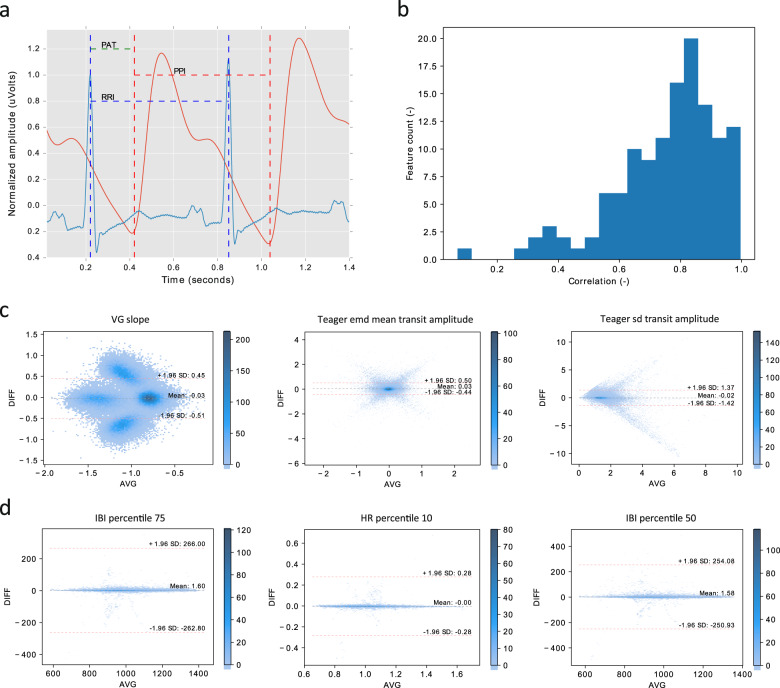
Discrepancy between ECG- and PPG-derived HRV features. **a** Inter-beat interval. A sequence of inter-beat intervals as recorded by ECG (blue, RR-interval, RRI) and PPG (red, peak-to-peak interval, PPI). Pulse arrival time (PAT) is also shown. **b** Distribution of correlations between ECG-derived and PPG-derived HRV features. The features were obtained from ECG and PPG signals simultaneously recorded in the Eindhoven data set. The detailed description of the features can be found in Supplementary Table 1. **c** Bland-Altman density plots of example features with low correlation. From left to right, the features are the slope of network degree distribution using a visibility graph method, the mean of inter-beat-interval series amplitudes (after empirical mode decomposition), and the standard deviation of inter-beat-interval series amplitudes at transition points detected based on a Teager energy method, with a correlation coefficient of 0.069, 0.264, and 0.308, respectively. **d** Bland-Altman density plots of example features with high correlation. From left to right, the features are the 75th percentile of inter-beat intervals, the 10th percentile of heart rates, and the 50th percentile of inter-beat intervals, with a correlation coefficient of 0.997, 0.995, and 0.994, respectively. AVG and DIFF indicate the mean and the difference between ECG and PPG feature values, respectively.

While both PPG and ECG measure heart beats and thus could be used for HRV measurement, there are two differences. The first is that the time delay between the heart contraction (ECG R-peak) and the arrival of the pulse at the wrist PPG, known as the pulse arrival time (Fig. 4a), is not constant: it is continuously modulated through properties of the arterial vessels such as blood pressure and vasoconstriction^35^, which change throughout the night^36^. Next to that, motion artefacts may impact the PPG sensor to an extent that heart beats cannot be extracted. These factors combined lead to HRV features being different when extracted from these sensors.

From HRV data computed from both ECG and PPG signals, a total of 127 features that have been proposed in our previous work^37,38^ were extracted. All features and their description are summarized in Supplementary Table 1. The HRV feature set had an average (Pearson’s) correlation of 0.76 ± 0.17 when derived from ECG versus PPG with a number of features having much lower correlation, as illustrated in Fig. 4b. While most features still retain a high (though not perfect) correlation between ECG and PPG, some of them are not correlated at all. As examples, the Bland-Altman density plots of three features with low and three features with high correlation between ECG and PPG are shown in Fig. 4c, d, respectively. In comparison with the high-correlation features, the low-correlation features show a much larger discrepancy between ECG- and PPG-derived feature values. We found that most low-correlation features are nonlinear features (such as visibility graph and Teager energy features) characterizing HRV time-series structures or dynamics, which are likely more sensitive to the differences between ECG- and PPG-derived inter-beat intervals or peak locations. On the other hand, most high-correlation features are statistical features in the time domain (such as percentiles of inter-beat intervals or heart rates) that are expected to be more robust to those differences or “outliers” in detected inter-beat intervals.

### Performance overview and comparison

Following the steps outlined in the training and validation schematic shown in Fig. 3, first the model was pre-trained on the entire Siesta data set for 1,472 training passes (determined through cross-validation) and evaluated on both the ECG and PPG signals of the Eindhoven data set. Cohen’s kappa for ECG was 0.62 ± 0.10 while accuracy was 74.83 ± 7.41%. For PPG data, Cohen’s kappa dropped to 0.57 ± 0.12 (*p* < 0.00001, Wilcoxon’s signed-rank test) and accuracy dropped to 71.88 ± 8.34% (*p* < 0.00001, Wilcoxon’s signed-rank test).

Subsequently, the model was re-initialized to random starting weights and trained in 4-fold cross-validation on the Eindhoven PPG data set, leaving out 15 participants for testing at each fold while training with the data of the remaining 45 participants. Cohen’s kappa and accuracy are shown in Table 2.

**Table 2:** Evaluation of different training strategies on the Eindhoven PPG data set.

Model	Training procedure summary	Cohen’s kappa	Accuracy (%)
ECG-trained model	Train^a^ on Siesta	0.57 ± 0.12	71.88 ± 8.34
PPG-trained model	Train^b^ on Eindhoven	0.55 ± 0.14	69.82 ± 10.23
Domain retrain	Pre-train^a^ on Siesta + adapt^b^ using Eindhoven	0.62 ± 0.12	75.21 ± 7.82
Decision retrain	Pre-train^a^ on Siesta + adapt^b^ using Eindhoven	0.63 ± 0.12	75.14 ± 8.10
Combined retrain	Pre-train^a^ on Siesta + adapt^b^ using Eindhoven	0.65 ± 0.11	76.36 ± 7.57

^a^Training was done on the entire Siesta ECG data set.

^b^Done in 4-fold cross-validation. In each fold 45 participants of the Eindhoven data set were used for training and 15 were left out for validation. Shown results are aggregated over all folds. All cross-validation experiments used the same folds to enable comparison. Results are presented as mean ± standard deviation. Distribution of performance over participants and statistical significance tests are shown in Fig. 5.

**Fig. 5:**
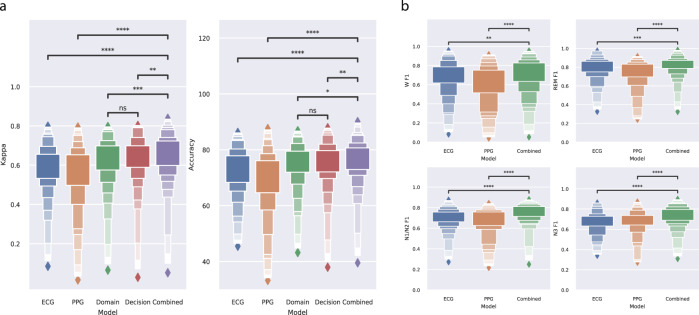
Comparison of sleep stage classification performance. **a** Evaluation of different model training strategies on the Eindhoven PPG data set. Distributions of participants in the data set are shown using letter value plots, in which box sizes are proportional to the number of participants in the box range. Performance reported in Cohen’s kappa and accuracy. **b** Comparison of the combined retrain transfer learning strategy with the two non-transfer baselines for each sleep stage, presented in F1 score. The same letter value plots and statistical testing annotation are used. Statistical comparisons between different models have been performed using Wilcoxon’s signed rank test (two-sided). Stars denote *p*-value of the test, where *, **, ***, and **** denote *p* < 0.05, *p* < 0.01, *p* < 0.001, and *p* < 0.0001, and “NS” denotes “not significant” or *p* > 0.05.

After determining the performance of the baselines, the three transfer learning approaches were evaluated. Cohen’s kappa and accuracy for the domain, decision, and combined retrain transfer learning approaches are given in Table 2, and the confusion matrix of the classifications obtained with the best model (combined retrain) is presented in Table 3. In Fig. 5a performance distributions are given for Cohen’s kappa and accuracy for the three transfer learning conditions as well as the two baseline conditions. Wilcoxon statistical test (two-sided) outcomes are also shown in the figure, revealing that the combined approach has the best average performance, and is significantly different from the other transfer learning approaches as well as both baselines.

**Table 3:** Confusion matrix of the combined retrain transfer learning model for sleep stage classification.

Predicted label → ↓ True label	Wake	N1/N2	N3	REM	Sensitivity (%)
Wake	8,468	2,588	199	885	69.75
N1/N2	2,670	31,449	4,332	3,347	75.24
N3	68	6,127	14,570	182	69.56
REM	461	2,298	103	14,690	83.69
PPV (%)	72.58	74.06	75.87	76.89	

Note: Results were obtained after classifying Wake, REM sleep, N1/N2 sleep, and N3 sleep in all epochs (*n* = 92,437) of all recordings on the Eindhoven PPG data set. Sensitivity and positive predictive value (PPV) for each class are presented.

The performance of the combined approach is also compared in terms of sleep stage specific F1 scores as illustrated in Fig. 5b. Wake (W) F1 was 0.71 ± 0.17 for the combined transfer model. This was significantly higher than for the ECG-trained (0.69 ± 0.17, *p* < 0.01) and PPG-trained (0.61 ± 0.20, *p* < 0.0001) Wake F1 scores. Analogously, REM F1 was 0.81 ± 0.10, significantly higher than ECG-trained (0.79 ± 0.11, *p* < 0.001) and PPG-trained (0.74 ± 0.14, *p* < .0001) REM F1. For N1/N2, the combined retrain F1 score was 0.75 ± 0.09, significantly higher than ECG-trained (0.70 ± 0.09, *p* < 0.0001) and PPG-trained (0.68 ± 0.12, *p* < 0.0001). Finally, for N3 the F1 score for the combined retrain model was 0.74 ± 0.09, which was also significantly higher than the N3 F1 score for ECG-trained (0.66 ± 0.10, *p* < 0.0001) and PPG-trained (0.68 ± 0.12, *p* < 0.0001) models.

**Fig. 6:**
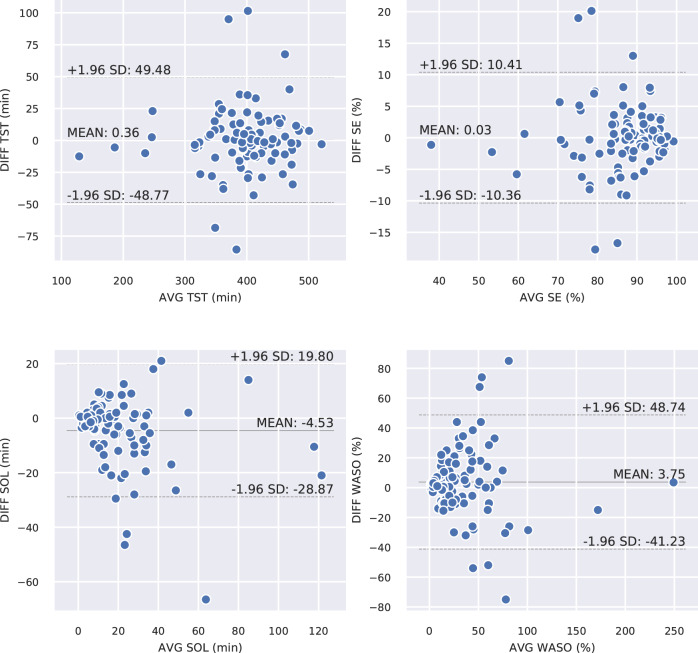
Bland-Altman analysis of the four main sleep-wake statistics, between reference and the combined retrain transfer learning approach. AVG on the horizontal axis is the mean between true and predicted values and DIFF on the vertical axis is the error (predicted value–true value).

Finally, Bland-Altman analysis for sleep-wake statistics TST, SE, SOL, and WASO are shown in Fig. 6 for the combined transfer approach, including mean error and 95% limits of agreement.

## Discussion

The main objective of this work was to evaluate a transfer learning approach to PPG-based sleep stage classification, in which a deep neural network model is first pre-trained using a large data set in a comparable domain and then parts of it are adapted for PPG. The data set used for pre-training was the Siesta data set in which ECG-based HRV features were available.

For the features with low correlation between ECG and PPG, certain patterns (clustering, symmetry, or trending) can be seen in the Bland-Altman density plots in Fig. 4c, suggesting the presence of systematic differences. As mentioned before, these can be caused by, for example, the known discrepancy in hemodynamic or cardiovascular physiology between HRV data measured from ECG and PPG signals and for assessing autonomic responses^39,40^, and a higher sensitivity of wrist-worn PPG to motion artefacts than ECG^41^. We hypothesize that these differences are learnable to a certain extent using transfer learning approaches.

It was shown first that the initially trained model for ECG indeed showed a higher performance on hold-out ECG data in comparison to PPG data that was extracted simultaneously. This confirms that the disparity between ECG- and PPG-derived features (see Fig. 4) also results in performance loss of a model trained for ECG. The LSTM model was also trained purely on the PPG data set, but the obtained performance did not differ significantly (Fig. 5a) from when the pre-trained ECG model is used.

Subsequently, the three transfer learning approaches were shown to significantly outperform the PPG-trained and the ECG-trained models, confirming that (1) adaptation to PPG of an ECG-based model can improve performance for PPG and (2) the PPG data set is not large enough to train such a deep model without pre-training.

The transfer learning approaches had, in addition to a better mean performance, also a slightly lower standard deviation (Fig. 5), suggesting that the model is more robust to unusual samples. In the case of the ECG-trained model, those could for example be people for which the ECG-derived and PPG-derived features were exceptionally different from each other. Those would result in low performance using that model in comparison to the transfer learning approaches where the model is adapted to PPG. Whereas for the PPG-trained model the unusual samples could be people with uncommon sleep architectures that the Eindhoven data set did not contain enough examples of for the model to learn about. By pre-training on the larger and more heterogenous Siesta data set the model became more robust to these unusual sleep architectures than it could have using only the smaller Eindhoven data set. A different reason for the performance improvement after transfer learning could also be the adaptation to the newer AASM annotation standard. The AASM rules result in structural differences in the annotation. The inter-rater agreement between human annotators using AASM rules has a Cohen’s kappa of 0.76 while agreement between annotators using R&K rules is only 0.68^42^.

Among the three transfer learning approaches, the combined approach achieved the highest performance (Table 2). In earlier transfer learning approaches^32,33^ only the softmax/decision layers of the model were retrained and other layers were never considered, however, this result implies that this is not always the optimal approach. More optimal retraining strategies may be found by considering also other layers. In comparison to previous work, the performance of the combined retrain transfer learning approach for PPG-based sleep stage classification is unprecedented. For 4-class sleep stage classification, Fonseca et al.^19^ evaluated a model that was previously trained using HRV features from a large ECG data set on a smaller PPG data set (51 healthy middle-aged adults) and a linear discriminant classifier, reporting a Cohen’s kappa of 0.42 for 4-class sleep stage classification. Wu et al.^22^ developed a support vector machine algorithm on a small PPG data set (31 healthy subjects), and reported a Cohen’s kappa of 0.41. Beattie et al.^20^ reported a Cohen’s kappa of 0.52 for a similar healthy demographic by training on a PPG data set and Fujimoto et al.^21^ achieved an accuracy of 68.8% using a similar method, also with healthy individuals (100). Recently, Korkalainen et al.^43^ performed sleep staging from PPG data obtained with a finger pulse oximeter instead of a wrist-worn device and achieved a slightly higher kappa of 0.54 and a similar accuracy of 68.5% in patients with suspected sleep apnoea. In our earlier work, we reported Cohen’s kappa of 0.56 using a clinical data set with a model trained on ECG data^44^, and it has been concluded that the direct application of an ECG-based model on PPG (without transfer learning) decreases the performance. The combined retrain transfer approach presented here outperformed all these works considerably with an average Cohen’s kappa of 0.65 and accuracy of 76.36%.

Because sleep statistics (derived from sleep-wake pattens) are vital measures of assessing sleep quality, we also compared our results with several studies that evaluated PPG for binary sleep-wake classification. For example, Uçar et al.^16^ reported an F1 score for wake of 0.79 in sleep apnoea patients, higher than obtained in this study (0.71) with healthy individuals, though they only used 10 individuals to evaluate the method, as compared to 60 individuals in this study. Walch et al.^18^ trained and validated their model (multilayer perceptron) using PPG data from Apple Watch (Apple Inc., Cupertino, CA) and achieved an accuracy of 90% (F1 score not reported). Haghayegh et al.^45^ reviewed studies using different Fitbit (Fibit Inc., San Francisco, CA) models in assessing sleep. They reported an overestimated TST of 7–67 min and SE of 2–15%, an underestimated WASO of 6–44 min, and a bias of 1–25 min for SOL, which, in general, worse than what we achieved in this work. Terjung et al.^17^ evaluated an algorithm for estimating sleep/wake statistics in patients suspected of sleep-disordered breathing. They reported higher bias ± [95% limit of agreement] for TST (14 ± [−82 to 54] min, versus 0.36 ± [−48.77 to 49.48] min in this work) and for SE (4 ± [−20 to 13.4]%, versus 0.03 ± [−10.36 to 10.41]% in this work). For WASO they reported a higher bias but smaller limits of agreement (−12 ± [−69.14 to 44.54] min, versus 3.75 ± [−31.23 to 48.74] min in this work) and for SOL estimation they reported smaller bias but higher limits of agreements (−1 min ± [−41 to 43] min, versus −4.53 ± [−28.87 to 19.80] min here). Trained on 70 and tested on 32 patients with sleep apnoea and/or periodic limb movement, Cakmak et al.^46^ reported a lower F1 score (0.62) with an higher bias for SE (2.09 ± [−3.97 to 8.15]%), for SOL (−22.86 ± [−44.01 to −1.7] min), and for WASO (7.66 ± [−16.62 to 31.94] min) compared with our results. As the population of the Eindhoven data set is healthy, it is difficult to conclusively compare with the data set used by Terjung et al. (which likely included many sleep-disordered patients) and the data set by Cakmak et al. The analyses in Fig. 6 show that estimation error for SOL and WASO tends to increase with unusually high SOL and WASO (which could be related to sleep insomnia). In addition, some other studies used fingertip PPG (pulse oximetry) data including both heart rate and oxygen saturation to detect sleep/wake state and achieved high performance^47,48^, but sleep statistics were not reported.

In the present work, we only considered 4-class sleep stage classification instead of classifying all 5 stages. It is known that the human inter-rater agreement is worst for N1 (considered a transitional stage) when performing PSG-based manual scoring, with a reported Cohen’s kappa of 0.46^42^. Moreover, when using autonomic data (such as HRV), detecting N1 is even more difficult, as evidenced by a very low detection rate (26.7%) and PPV (3.7%) as reported in our previous work^38^. This is likely because changes in autonomic nervous activity are in nature slower than cortical changes and N1 sleep is much shorter than the other sleep stages, making those changes hardly visible in HRV features during N1 sleep. Thus, like many other studies, we merged N1 and N2 as one single class (often called light sleep).

It is known that sleep disordered breathing events (such as obstructive sleep apnoea and hypopnoea) disturb sleep architecture^49^. It is therefore important to understand how the algorithm performs in patients with different severity classifications of sleep apnoea as measured by apnoea-hypopnoea index (AHI). For the Siesta data set, AHI was only available from automatic scoring using the Somnolyzer auto-scoring software (Philips Respironics, Murrysville, PA), not verified manually. Therefore, we decided to not present this information and avoid possibly misleading results. For the Eindhoven data set, AHI is not available because only the minimum set of PSG channels needed to score sleep stages was used, and two of the essential respiratory channels (airflow and SpO2) were not recorded. Future studies are required to analyse the effect of sleep apnoea/hypopnoea on our proposed algorithm in sleep stage classification.

This study used wrist-worn reflective PPG instead of finger PPG that is routinely used in clinics, mainly because of its ease of use and capability of measuring heart beats (pulses) during sleep, making it an attractive sensing modality for monitoring sleep at home. The reflective PPG device provides only one optical wavelength (green light), and is therefore not adequate to measure oxygen saturation or SpO2 and therefore assess sleep disordered breathing. Moreover, the PPG signal measured using green light (corresponding to a shorter wavelength) has a higher signal-to-noise ratio, allowing for higher accuracy in pulse detection and HRV measurement compared with red and infrared^50^. Despite these advantages, it is worth further exploring the feasibility of adding SpO2 for improving sleep staging performance for sleep-disordered patients and detecting sleep disordered breathing. Previous studies have shown promising results in sleep staging by combining HRV with accelerometer data^20,21^. Yet this was not possible in our work when using transfer learning approaches because there was no accelerometer data available in the Siesta data. Future work should investigate methods allowing accelerometer data to be added to the model, while retaining the HRV part trained based on transfer learning.

Another limitation of this work is, as shown in Table 1, that the age range in the Eindhoven data set (44–60 years) is limited in comparison with the Siesta data set, and that all subjects from this data set were healthy (and therefore did not have any sleep disorders). Since the present work focused on the validation of our proposed method in health subjects, future work in which sleep disordered individuals are included could make the method more robust to unusual samples such as these. Given the large performance improvement achieved with the transfer learning approach for healthy individuals compared to non-transfer approaches, we anticipate that the method will also lead to improvements in sleep-disordered populations.

In conclusion, a transfer learning approach has been proposed for wearable sleep stage classification using a PPG sensor and an LSTM deep neural network. The proposed transfer learning strategy was compared to two baselines: training the model on a large ECG data set, as well as training the model only on the (smaller) amount of PPG data available. It was shown that transfer learning outperforms both approaches, suggesting that deep temporal knowledge can be generalized over different sensor modalities.

## Methods

### Data sets and procedure

The Siesta data set was collected as part of the EU Siesta project^34^ in the period from 1997 to 2000 in seven European countries. The study was approved by the local ethical committee of each research group (e.g., Medical University of Vienna, Austria) and all participants signed informed consent. Participants had no history of alcohol or drug use or worked shifts. The data set includes 195 healthy participants and 97 patients with a sleep or sleep-disturbing disorder (26 patients with insomnia, 51 with sleep apnoea, 5 with periodic limb movement disorder, and 15 with Parkinson’s disease). Each participant underwent two nights of PSG monitoring in a sleep lab. The PSG included EEG, ECG, electrooculography (EOG), and electromyography (EMG) measurements. Each recording was scored by two trained somnologists from different sleep centres according to the R&K guidelines^9^, and revised by a third expert who took the final decision in case of disagreement. For a small portion of the data set, the data was also scored according to AASM guidelines, which were used to create the example illustrated in Fig. 1. A major aim of Siesta was to create a large normative sleep database of both healthy subjects and patients with sleep disorders across all age groups from 20 to >80. More details regarding participants and study design were described by Klosh et al.^34^

The second data set was collected in 2014 and 2015 in Eindhoven, the Netherlands, approved by the Internal Committee of Biomedical Experiments of Philips Research and conducted in accordance with the Declaration of Helsinki. Before participation, all participants gave informed consent. It includes 101 recordings of 60 healthy participants with no primary history of neurological, cardiovascular, psychiatric, pulmonary, endocrinological, or sleep disorders. In addition, none of the participants were using sleep, antidepressant or cardiovascular medication, recreational drugs or excessive amounts of alcohol. Each of the participants underwent one or two nights of PSG measurements in a hotel including EEG, ECG, EMG, and EOG used to annotate the epochs according to the AASM sleep scoring rules^8^. Next to the standard PSG system, also a CE-marked logging device containing a PPG and a tri-axial accelerometer sensor (Royal Philips, Amsterdam, the Netherlands) was used. The logging device was mounted on the non-dominant wrist of the participant, with the sensor facing the skin on the dorsal side of the hand, above the ulnar styloid process. The PSG data were annotated by a trained sleep technician according to the AASM rules of sleep scoring^8^. The aim of creating this data set was to collect wrist-worn PPG data with simultaneously recorded PSG as gold standard for developing PPG-based sleep monitoring algorithms. More details regarding study design were described in the work by Fonseca et al.^19^

### HRV feature extraction

The ECG signals of both the Eindhoven and Siesta data sets, as well as the PPG signals of the Eindhoven data set, were processed by beat detection algorithms to extract series of inter-beat intervals for each night of data. For ECG, the algorithm was a modification^51^ of the Hamilton-Tompkins beat detection^52^. For PPG signals, the algorithm described by Papini et al.^53^ was used. From these inter-beat-interval series, a set of 127 HRV features were extracted. This extensive set of sleep features has been well-documented and motivated in our earlier work^37,38^. It includes 37 time domain statistical features, 14 frequency-domain features, 8 phase coordination features, 28 features measuring entropy and self-similarity of the heart beat series, 22 features in the Teager energy domain, 5 statistical features of cortical sleep arousal likelihood and 13 features based on visibility graph analysis (see Supplementary Table 1 for feature descriptions and corresponding references). Each feature value *f* was normalised through $f_n = \frac{{f^p - \mu }}{\sigma }$. The parameters *p*, *μ*, and *σ* are determined during training using the entire training set. *p* was chosen to maximize the feature’s distribution similarity to a uniform distribution, and on this new distribution the mean (*μ*) and standard deviation (*σ*) were determined.

### The LSTM model

**Fig. 7:**
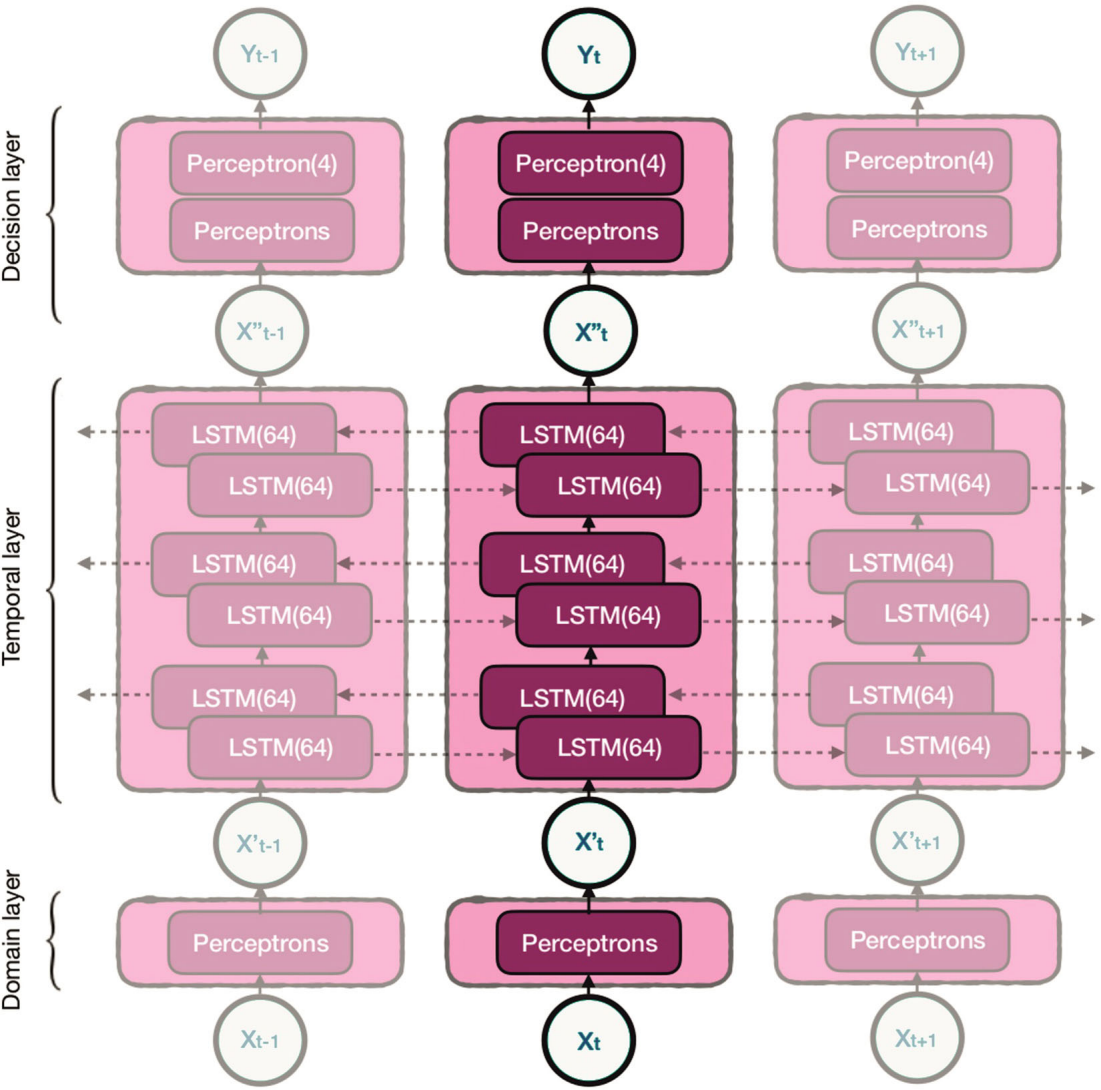
A partially rolled-out overview of the neural network architecture to visualise the temporal interaction. The dotted arrows indicate the flow direction of temporal information through LSTM connections.

A single model architecture was used throughout all experiments. The model, illustrated in Fig. 7, is conceptually divided into three blocks:

*Domain layer* consists of perceptrons that make 32 linear combinations of features. The function of this layer is to pre-weight, pre-select and combine the input into a more compact representation of the domain: a vector of 32 values per time-step. We call this output *X*′ as it is a translation of *X*. As this vector is generated for each time step *t*, the domain layer generates a sequence of vector $\{ X_1^\prime , \ldots ,X_n^\prime \}$, with $|X_t^\prime | = 32$.

*Temporal layer* consists of the LSTM stacks. These LSTMs take the sequence $\{ X_1^\prime , \ldots ,X_n^\prime \}$ and generate 128 new features at each time step, where temporal information has been taken into account through the short- and long-term recurrence properties both from the past and future. This results in a sequence of feature vectors $\{ X_1^\prime\prime , \ldots ,X_n^\prime\prime \}$, where ._._.

*Decision layer* consists of two levels of perceptrons. The first level performs a dimensionality reduction of the output of the temporal layer, reducing the vector of size 128 to size 32. The final level contains 4 softmax perceptrons: each perceptron generates a sigmoid output representing the 4 class probabilities $P(Y_t = W|X_t^\prime\prime )\prime\prime$ $P(Y_t = R|X_t^\prime\prime )\prime\prime$ $P(Y_t = N1/N2|X_t^\prime\prime )\prime\prime$ and $P(Y_t = N3|X_t^\prime\prime )\prime\prime$. The outputs sum up to one for each time step through the softmax normalisation. These outputs are generated for each time step, resulting in a sequence of sleep stage probabilities corresponding to all epochs of the entire night.

The 2.6 × 10^5^ free parameters of the model were trained simultaneously with the RMSprop optimizer^54^. Dropout^55^ on the input (20%), on LSTM outputs (50%) and recurrent LSTM connections^56^ (50%) was applied during each training phase to reduce overfitting. Categorical cross-entropy was used as the loss function during model fitting.

### Baseline model training and evaluation

A number of models are trained and compared with the goal of validating the LSTM model on ECG data and comparing three transfer learning methods against two conventional learning methods. The entire validation scheme is illustrated in Fig. 3 through numbered blocks.

First, the model is pre-trained on the Siesta data set. The inputs are the HRV features computed from ECG and the labels are the R&K annotations. The validation is done in a 4-fold cross-validation scheme in which folds are created on participant level, thus ensuring that nights from the same participants are always either in the training or the testing portion. An early stopping criterion is used that stops training the model once the loss on the test fold does not improve for 100 subsequent passes over the training data. Subsequently, the model is trained on the entire Siesta data set to generate the *source model*, which will be referred to as the *ECG model* (block 2 in Fig. 3). The number of epochs is taken as the average number of epochs until early stopping across the four folds in the earlier step.

This model is then used without any adaptation to classify the entire Eindhoven data set using two different inputs. First, the ECG recordings of the Eindhoven data set are used as input as these are a hold-out that was not used in creating the ECG model (block 3 in Fig. 3). Then, the model is also used without adaptation to classify the Eindhoven PPG data as a means to quantify the performance drop due to a change in sensing modality (block 4 in Fig. 3). This forms the first baseline for PPG-based sleep stage classification.

The conventional way in which the model would be trained for PPG-based sleep stage classification is by training only using PPG data. This is the second baseline condition. The LSTM model is trained using the PPG sequences of the Eindhoven data set as inputs and the AASM annotations as labels. This is done using 4-fold cross-validation and the predictions over all folds are used to compute the performance of this model (block 5 in Fig. 3).

### Transfer learning conditions and evaluation

As mentioned, the model was conceptually divided into three components. The temporal component contains the deep temporal representation of sleep physiology in relation to sleep stage progression. This part is expected to generalize to different sensors as well as changes in annotation standard. This layer also contains 96.6% of the model’s weights, making it particularly hard to train on smaller data sets. These reasons make the temporal layer a good candidate for transfer as only a small amount of weights would need to be adapted using the scarce target data set. Thus, the candidate layers for retraining are the domain and decision layers. Retraining the domain layer would imply adapting the domain layer to make *X*′ more comparable to what the ECG model expects (Fig. 7), while alternatively retraining the decision layer implies adapting the mapping between *X*″ and the labels. The third option is to retrain both domain and decision layers simultaneously, leaving it up to the optimizer to change weights as needed in both of the layers.

All three strategies are tested by freezing all layers of the source model and then retraining the respective layers using 4-fold cross-validation (blocks 6, 7, and 8 in Fig. 3) as used for the PPG-trained baseline. The fold splitting was kept constant across all experiments. When a layer is frozen, its weights will not be updated during training anymore. However, the dropout mechanism remains in place: connections in the temporal layer will be randomly omitted to improve generalisability during training passes, even though the weights associated with these connections will not be changed. The models created with these three transfer learning strategies will be referred to as the domain, decision, and combined models, referring to which layers are retrained in the condition.

### Analysis of performance

The main measures of performance were accuracy and Cohen’s kappa. First, the performance of the ECG model on the Eindhoven ECG versus the Eindhoven PPG data is compared to understand the loss in performance due to the domain shift from ECG to wearable PPG. These are the outcomes of blocks 3 and 4 in Fig. 3. Then, the three transfer learning strategies (*domain*, *decision*, and *combined*; block 6, 7, and 8 in Fig. 3) are compared in terms of their performance on Eindhoven PPG data to understand which strategy leads to the best knowledge transfer. The best transfer strategy is then compared to the performance of the two baseline models on PPG, namely the pre-trained ECG model (block 4 in Fig. 3) and the PPG-only model (block 5 in Fig. 3). This is done to show that the transfer learning method outperforms non-transfer baselines. For this comparison, performance is also compared in terms of the F1 score for each of the 4 sleep stage classes (W, REM, N1/N2, and N3). Since all evaluations are performed on the Eindhoven PPG data set (with the same participants), a paired statistical comparison is performed with performance measured per participant. The chosen test was the Wilcoxon’s signed-rank test (two-sided), as it does not assume normality of the performance distributions.

For the best-performing model, a confusion matrix was presented. In addition, the sleep-wake statistics TST (total non-waking time in the night), SE (percentage of non-waking time of the night), WASO (amount of time awake after first sleep epoch and before last sleep epoch), and SOL (time spent awake before falling asleep at the start of the night) were computed. Comparison with sleep statistics derived from reference hypnogram was done using Bland-Altman analysis^57^.

### Reporting summary

Further information on research design is available in the Nature Research Reporting Summary linked to this article.

## Supplementary information

Supplementary Information

Reporting Summary

## Data Availability

The data used in this study are not publicly available. The Siesta data were obtained from the EU Siesta project; data are available upon reasonable request to the Siesta group (The Siesta Group Schlafanalyse GmbH, http://www.thesiestagroup.com). Specific restrictions apply to the availability of the Eindhoven data collected with sensors used under license. These data are however available from the authors upon reasonable request and with permission of the licensors and compliance to The General Data Protection Regulation (GDPR) in the EU.

## Code availability

The codes used in the study are proprietary, and available only with the permission of the licensors.
