# Supplementary material for: A deep transfer learning approach for wearable sleep stage classification with photoplethysmography
Source: NPJ Digit Med. 2021 Sep 15;4:135. doi: 10.1038/s41746-021-00510-8 (PMC8443610; doi:10.1038/s41746-021-00510-8)
Supplement: Supplementary file 1 — Supplementary Information [file 41746_2021_510_MOESM1_ESM.pdf]

Supplementary Table 1. Summary of all 127 HRV features used in the work.

Count	Feature
**37**	**Time domain features**
5	Mean HR, mean RR, detrended mean RR, and max and min RR[S1,S2]
10	SDNN, SDRR, RR range, pNN50, RMSSD, and SDSD, and MAD (both RR and detrended RR)[S1,S3]
22	Percentiles (5%, 10%, 25%, 50%, 75%, 90%, and 95%) of RR, percentiles (10%, 25%, 50%, 75%, and 90%) of HR, detrended HR, and detrended RR[S3]
**14**	**Frequency domain features**
4	RR logarithmic VLF, LF, and HF band power and LF-to-HF ratio[S1,S4]
6	Boundary-adapted RR logarithmic VLF, LF, and HF band power, LF peak and its power, LF-to-HF ratio[S5]
4	RR mean respiratory frequency and power, max phase and module in HF pole[S6]
**8**	**Phase coordination features**
4	Phase synchronization for 6:2, 7:2, 8:2 and 9:2 ratios of RR intervals[S7,S8]
3	Dominant ratio, short- and long-term phase coordination of RR intervals[S7,S8]
1	Higuchi's fractal dimension of the phase coordination of RR intervals[S9]
**28**	**Entropy and self-similarity features**
20	Multiscale sample entropy of RR intervals at length 1 and 2, scales 1-10[S10]
1	Sample entropy of symbolic binary changes in RR intervals[S11]
7	RR DFA, its short, long exponents, and all scales, DFA using Peng's method, WDFA and PDFA[S12,S13,S14]
**22**	**Teager energy features**
11	On RR series: mean Teager energy, % of transition points and local maxima, mean and SD of intervals between consecutive transition points and distances between local maxima points, mean and SD of RR amplitudes at transition points and local maxima values[S15,S16]
11	On IMF1 of RR series: mean Teager energy, % of transition points and local maxima, mean and SD of intervals between consecutive transition points and distances between local maxima points, mean and SD of RR amplitudes at transition points and local maxima values[S15,S16]
**13**	**Visibility graph features**
6	Mean and SD of degrees and clustering coefficients of VG and difference-VG networks[S17,S18]
6	Slope of power-law fit to degree distribution, % of nodes with small and high degree of VG and difference-VG networks[S17]
1	Assortativity mixing coefficient of VG network[S17]
**5**	**Arousal features**
5	Max, mean, median, min and SD of arousal probabilities[S19]

HR: heart rate, RR: R-R intervals (for ECG) or inter-beat intervals (peak-to-peak intervals for PPG), SDNN: standard deviation of normal-to-normal (NN) beat intervals, SDRR, standard deviation of RR intervals, pNN50: percentage of successive RR differences, RMSSD: root mean square of successive RR differences, SDSD: standard deviation of successive RR differences, MAD: mean absolute difference, VLF: very low frequency, LF: low frequency, HF: high frequency, DFA: detrended fluctuation analysis, PDFA: progressive DFA, WDFA: windowed DFA, IMF1: the first intrinsic mode function of empirical mode decomposition, SD: standard deviation, VG: visibility graph. The choice of parameters and window size to compute epoch-based feature values has been discussed in our earlier work[S20,S21].

References

S1. Task Force of the European Society of Cardiology and the North American Society of Pacing and Electrophysiology. Heart rate variability: standards of measurement, physiological interpretation and clinical use. *Circulation* **93**, 1043–1065 (1996).

S2. Redmond, J. & Heneghan, C. Cardiorespiratory-based sleep staging in subjects with obstructive sleep apnea. *IEEE Trans. Biomed. Eng.* **53**, 485–496 (2006).

S3. Yılmaz, B., Asyalı, M. H., Arıkan, E., Yetkin, S. & Özgen, F. Sleep stage and obstructive apneaic epoch classification using single-lead ECG. *Biomed. Eng. Online* **9**, 39 (2010).

S4. Bušek, P., Vaňková, J., Opavský, J., Salinger, J. & Nevšímalová, S. Spectral analysis of the heart rate variability in sleep. *Physiol. Res*. **54**, 369–376 (2005).

S5. Long, X., Fonseca, P., Haakma, R., Aarts, R. M. & Foussier, J. Spectral boundary adaptation on heart rate variability for sleep and wake classification. *Int. J. Artif. Intell. Tools* **23**, 1460002 (2014).

S6. Mendez, M. O. et al. Sleep staging from heart rate variability: time-varying spectral features and hidden markov models. *Int. J. Biomed. Eng. Technol.* **3**, 246–263 (2010).

S7. Bettermann, H., Cysarz, D. & Van Leeuwen, P. Detecting cardiorespiratory coordination by respiratory pattern analysis of heart period dynamics - the musical rhythm approach. *Int. J. Bifurc. Chaos* **10**, 2349–2360 (2000).

S8. Cysarz, D., Bettermann, H., Lange, S., Geue, D. & van Leeuwen, P. A quantitative comparison of different methods to detect cardiorespiratory coordination during night-time sleep. *Biome. Eng. Online* **3**, 44 (2004).

S9. Higuchi, T. Approach to an irregular time series on the basis of the fractal theory. *Physica D* **31**, 277–283 (1988).

S10. Costa, M., Goldbeerger, A. & Peng, C.-K. Multiscale entropy analysis of complex physiologic time series. *Phys. Rev. Lett*. **89**, 068102 (2002).

S11. Cysarz, D., Bettermann, H. & van Leeuwen, P. Entropies of short binary sequences in heart period dynamics. *Am. J. Physiol. – Heart Circ. Physiol*. **278**, 2163–2172 (2000).

S12. Kantelhardt, J. W., Koscielny-Bunde, E., Rego, H. H. A., Havlin, S. & Bunde, A. Detecting long-range correlations with detrended fluctuation analysis. *Physica A* **295**, 441–454 (2001).

S13. Penzel, T., Kantelhardt, J. W., Grote, L., Peter, J.-H. H. & Bunde, A. Comparison of detrended fluctuation analysis and spectral analysis for heart rate variability in sleep and sleep apnea. *IEEE Trans. Biomed. Eng.* **50**, 1143–1151 (2003).

S14. Telser, S. et al. Can one detect sleep stage transitions for on-line sleep scoring by monitoring the heart rate variability? *Somnologie* **8**, 33–41 (2004).

S15. Kvedalen, E. Signal processing using the Teager Energy Operator and other nonlinear operators (University of Oslo, 2003).

S16. Yang, J., Long, X. & Haakma, R. Feature extraction for deep sleep detection. Technical Note, PR-TN 2013/00405 (Philips Research, 2013).

S17. Long, X., Fonseca, P., Aarts, R. M., Haakma, R. & Foussier, J. Modeling cardiorespiratory interaction during human sleep with complex networks. *Appl. Phys. Lett.* **105**, 203701 (2014).

S18. Zhu, G., Li, Y. & Wen, P. P. Analysis and classification of sleep stages based on difference visibility graphs from a single-channel EEG signal. *IEEE J. Biomed. Health Inform*. **18**, 1813–1821 (2014).

S19. Basner, M., Griefahn, B., Müller, U., Plath, G. & Samel, A. An ECG-based algorithm for the automatic identification of autonomic activations associated with cortical arousal. *Sleep* **30**, 1349–1361 (2007).

S20. Fonseca, P. et al. Sleep stage classification with ECG and respiratory effort. *Physiol. Meas*. **36**, 2027–2040 (2015).

S21. Fonseca, P., den Teuling, N., Long, X. & Aarts R. M. Cardiorespiratory sleep stage detection using conditional random fields. *IEEE J. Biomed. Health Inform*. **21**, 956–966 (2017).
