# Supplementary material for: A deep transfer learning approach for wearable sleep stage classification with photoplethysmography
Source: NPJ Digit Med. 2021 Sep 15;4:135. doi: 10.1038/s41746-021-00510-8 (PMC8443610; doi:10.1038/s41746-021-00510-8)
Supplement: Supplementary file 2 — Reporting Summary [file 41746_2021_510_MOESM2_ESM.pdf]

nature portfolio

Corresponding author(s): Xi Long
Last updated by author(s): Jul 6, 2021

Reporting Summary

Nature Portfolio wishes to improve the reproducibility of the work that we publish. This form provides structure for consistency and transparency in reporting. For further information on Nature Portfolio policies, see our Editorial Policies and the Editorial Policy Checklist.

Statistics

For all statistical analyses, confirm that the following items are present in the figure legend, table legend, main text, or Methods section.

n/a	Confirmed	
☐	☒	The exact sample size (*n*) for each experimental group/condition, given as a discrete number and unit of measurement
☐	☒	A statement on whether measurements were taken from distinct samples or whether the same sample was measured repeatedly
☐	☒	The statistical test(s) used AND whether they are one- or two-sided<br>*Only common tests should be described solely by name; describe more complex techniques in the Methods section.*
☐	☒	A description of all covariates tested
☐	☒	A description of any assumptions or corrections, such as tests of normality and adjustment for multiple comparisons
☐	☒	A full description of the statistical parameters including central tendency (e.g. means) or other basic estimates (e.g. regression coefficient) AND variation (e.g. standard deviation) or associated estimates of uncertainty (e.g. confidence intervals)
☐	☒	For null hypothesis testing, the test statistic (e.g. *F*, *t*, *r*) with confidence intervals, effect sizes, degrees of freedom and *P* value noted<br>*Give P values as exact values whenever suitable.*
☒	☐	For Bayesian analysis, information on the choice of priors and Markov chain Monte Carlo settings
☒	☐	For hierarchical and complex designs, identification of the appropriate level for tests and full reporting of outcomes
☒	☐	Estimates of effect sizes (e.g. Cohen's *d*, Pearson's *r*), indicating how they were calculated

Our web collection on statistics for biologists contains articles on many of the points above.

Software and code

Policy information about availability of computer code

Data collection	The Siesta data used in this manuscript were collected as part of the EU SIESTA project in the period from 1997 to 2000 (http://www.ofai.at/siesta/database), stored as EDF files on CD-ROMs. The data are available upon reasonable request to the Siesta Group Schlafanalyses GmbH (http://www.thesiestagroup.com). The Eindhoven data used in this manuscript were collected by using the the Philips Respironics Alice PDx system bundled with the Sleepware software (Philips Respironics Inc., Murrysville, PA, USA) and a CE-marked logging device containing a PPG and accelerometer sensors (Royal Philips, Amsterdam, the Netherlands).
Data analysis	For scoring some sleep-related events (Eindhoven data), the computer-supported scoring (software) system Somnolyzer (Philips Respironics Inc., Murrysville, PA, USA) was used; and for scoring sleep stages (Eindhoven data), the PSG data were analyzed by an external experienced somnologist (SleepVision, Nijmegen, the Netherlands). Data were analyzed to train and validate sleep stage classification models using our own custom built algorithms. The algorithms used several open-source packages (from, e.g. Python, TensorFlow/Keras). The core algorithms (such as feature extraction, LSTM modelling) have been described in published literature. The codes used in the study are proprietary, and available only with the permission of the licensors.

For manuscripts utilizing custom algorithms or software that are central to the research but not yet described in published literature, software must be made available to editors and reviewers. We strongly encourage code deposition in a community repository (e.g. GitHub). See the Nature Portfolio guidelines for submitting code & software for further information.

Data

Policy information about availability of data

All manuscripts must include a data availability statement. This statement should provide the following information, where applicable:

- Accession codes, unique identifiers, or web links for publicly available datasets
- A description of any restrictions on data availability
- For clinical datasets or third party data, please ensure that the statement adheres to our policy

The data used in this study are not publicly available. The Siesta data were obtained from the EU Siesta project; data are available upon reasonable request to the Siesta Group (The Siesta Group Schlafanalyse GmbH, http://www.thesiestagroup.com). Specific restrictions apply to the availability of the Eindhoven data collected with sensors used under license. These data are however available from the authors upon reasonable request and with permission of the licensors and compliance to The General Data Protection Regulation (GDPR) in the EU.

Field-specific reporting

Please select the one below that is the best fit for your research. If you are not sure, read the appropriate sections before making your selection.

☒ Life sciences ☐ Behavioural & social sciences ☐ Ecological, evolutionary & environmental sciences

For a reference copy of the document with all sections, see nature.com/documents/nr-reporting-summary-flat.pdf

Life sciences study design

All studies must disclose on these points even when the disclosure is negative.

Sample size	For training the model, we included data obtained from the EU Siesta project (in seven European countries), which aimed to create a large normative database of both healthy subjects and patients with sleep disturbances of disorders affecting their sleep. It includes two-night PSG recordings from around 200 normals cross all age groups and data from up to 100 subjects with disorders (such as sleep apnea, insomnia, Parkinson's disease, and PLMS). The goal of using this large cohort data set was to facilitate the training of a reliable LSTM model with sufficient diversity in samples (in terms of, e.g., age, gender, healthy subjects/sleep disorders) for applying transfer learning for sleep staging and evaluate the model on a targeted held-out population.
Data exclusions	Data were excluded (2 out of 294 subjects) because of invalid PSG measurements or sleep hypnogram annotation.
Replication	The model was evaluated on data not included for model training, or we performed cross validation in subject-level (where data from the same subjects were never included in both training/transfer-learning and test data set).
Randomization	Training was based on all the available data from the Siesta data set. For transfer learning and testing (on the Eindhoven data), subjects for were randomly split into four folds, as detailed in the manuscript for subject-level cross validation.
Blinding	Blinding was not applicable based on the design of our study.

Reporting for specific materials, systems and methods

We require information from authors about some types of materials, experimental systems and methods used in many studies. Here, indicate whether each material, system or method listed is relevant to your study. If you are not sure if a list item applies to your research, read the appropriate section before selecting a response.

Materials & experimental systems

n/a	Involved in the study	
☒	☐	Antibodies
☒	☐	Eukaryotic cell lines
☒	☐	Palaeontology and archaeology
☒	☐	Animals and other organisms
☐	☒	Human research participants
☒	☐	Clinical data
☒	☐	Dual use research of concern

Methods

n/a	Involved in the study	
☒	☐	ChIP-seq
☒	☐	Flow cytometry
☒	☐	MRI-based neuroimaging

Human research participants

Policy information about studies involving human research participants

Population characteristics	For the Siesta data set, there are 292 participants (195 healthy individuals and 97 patients with insomnia, sleep apnea,

Population characteristics	periodic limb movement disorders or Parkinson's disease; 126 females and 166 males). They had a mean (standard deviation) age of 51.5 (17.3) years (range: 20-95 years) and a mean (standard deviation) BMI of 25.6 (4.5) kg/m2 (range: 16.5-43.3 kg/m2).<br>For the Eindhoven data set, there are 60 participants (healthy individuals, 26 females and 34 males). They had a mean (standard deviation) age of 51.7 (7.9) years (range: 41-66 years) and a mean (standard deviation) BMI of 25.6 (3.9) kg/m2 (range: 17.5-36.2 kg/m2).
Recruitment	The Siesta data were obtained from the EU SIESTA project, in seven European countries. For the Eindhoven data, participants were recruited for recording two-night PSG and PPG in a hotel and the recruitment focused on healthy middle-aged participants.
Ethics oversight	For the Siesta data, study protocol was approved by the local ethical committee of each research group (e.g., Medical University of Vienna, Austria) and all participants signed informed consent. For the Eindhoven data, study protocol was approved by the Internal Committee of Biomedical Experiments of Philips Research and conducted in accordance with the Declaration of Helsinki, and all participants provided informed consent.

Note that full information on the approval of the study protocol must also be provided in the manuscript.
